# The Role of the Surgeon and Transurethral Resection in the Treatment of Superficial Bladder Cancer

**DOI:** 10.1100/tsw.2006.405

**Published:** 2006-07-06

**Authors:** Alan M. Nieder, Murugesan Manoharan

**Affiliations:** Department of Urology, University of Miami Miller School of Medicine, Miami, FL, USA

**Keywords:** superficial bladder cancer, transurethral resection

## Abstract

Non-muscle invasive bladder cancers are a heterogeneous group of cancers whose spectrum includes low grade Ta lesions and high-grade T1 lesions. Accurate staging and grading during initial evaluation and TUR ensures appropriate treatment and prevents the risk of understaging. TUR should be ideally performed under spinal anesthesia, with a continuous flow video resectoscope to maintain a stable bladder capacity, and a video monitor. The entire bladder must be visualized, with both 30- and 70-degree lenses, and all abnormal areas must be resected, with separate biopsies from each tumor's base. Repeat TUR is recommended for all high grade tumors and T1 tumors, especially if muscle was not present in the initial specimen. Immediate instillation of single dose chemotherapy agents following TUR is highly recommended to reduce the risk of tumor recurrences.

## INTRODUCTION

In 2006, over 60,000 Americans will be diagnosed with bladder cancer and over 12,000 patients will die from the disease[1]. Superficial urothelial carcinoma of the bladder refers to the tumor confined to the mucosa (Ta and TIS) or submucosa (T1). This is a heterogeneous disease with a variable natural history. At one end of the spectrum, low-grade Ta tumors have a low progression rate and rarely are a threat to the patient's life. On the other hand, high-grade T1 diseases have a high malignant potential with a significant progression and cancer death rates. Hence, it is disputed whether these should be considered as "superficial" disease. We have previously suggested that the term "superficial bladder cancer" should be abolished[2]. Seventy percent of the bladder tumors present as superficial disease. Approximately 70% of these tumors present as Ta disease, 20% as T1, and 10% as carcinoma *in situ*. The treatment and prognosis of bladder cancer varies significantly depending on the stage and grade of the disease. Hence it is critical to stage and grade the disease accurately.

Transurethral resection (TUR) is the key diagnostic modality to determine whether patients have bladder cancer that can be treated locally or requires more aggressive, surgical treatment. While TUR is an extremely common urologic procedure, it is not without complications[3]. Furthermore, by standardizing and maximizing the surgical technique of TUR and its indications, patient morbidity and potential mortality can be significantly reduced. Recent international guidelines have attempted to standardize the procedure of TUR and we will highlight some of these techniques[4,5,6].

## EVALUATION OF BLADDER CANCER

All patients with gross hematuria and those with microscopic hematuria — according to the American Urologic Association Best Practice Guidelines — should undergo a full urologic evaluation[7]. This workup consists of an upper-tract imaging study (e.g., Computerized Tomography scan without and with intravenous contrast or Intravenous Pyelogram), cystoscopy, urinalysis, and urine cytology. While bladder cancer tends to occur most frequently in older men, it is not uncommon for younger men and women to be diagnosed with the disease. In fact, it is often the youngest patients who present with the greatest delay in diagnosis[8]. Women with microscopic hematuria and irritative voiding symptoms (i.e., frequency, urgency, dysuria) are often treated with multiple courses of empiric antibiotics for suspected urinary tract infections[9]. Conversely, men with microscopic hematuria and irritative symptoms are often empirically treated for an enlarged prostate with alpha blockers. Moreover, patients on anticoagulation are often referred late for an evaluation since their bleeding is often attributed to their being anticoagulated.

## OFFICE CYSTOSCOPY

Cystoscopy has evolved considerably over the last 20 years from a painful evaluation with a rigid endoscope to a better-tolerated examination with a flexible endoscope. Contemporary flexible cystoscopy can be performed easily and quickly in the office with only lidocaine jelly as a topical anesthetic. Besides comfort, flexible cystoscopy has other advantages over rigid cystoscopy: It is easy to perform in the office and thus spares the expense of an operating room facility charge; anesthetic requirements are minimal; and since the patient is in a supine position when the procedure is performed, the time and expense of positioning and draping the patient is reduced. It provides excellent visualization of the entire bladder. Furthermore, for patients with a history of recurrent, noninvasive, low-grade urothelial tumors, we are able to biopsy and fulgurate small papillary lesions in the office. Most patients easily tolerate office fulguration with only periurethral lidocaine as an anesthetic.

Another revolution in cystoscopy has recently occurred with the introduction of digital cystoscopes. These scopes have only one plug-in for both camera image and light source, thereby simplifying setup. High-quality digital images can be obtained, and they can be either directly printed or stored onto a computer. In our practice, we share these images with the patient and they are saved in the patient's chart. The images are also reviewed with our trainees to demonstrate and teach the appearance of high- and low-grade lesions.

## TECHNIQUE OF TRANSURETHRAL RESECTION

Transurethral resection of bladder tumors (TURBT) is one of the most common procedures urologists perform, often on a daily basis. At the University of Miami, we have created a standard protocol for teaching our house staff residents on the optimal performance of TUR (Table 1). By instructing our house staff in these techniques, we believe that the outcomes can be maximized and morbidity can be minimized.

**TABLE 1**
**Optimal Technique of TUR**

- Spinal anesthetic
- Bimanual evaluation prior to and after resection
- Complete cystoscopic examination with both 30- and 70- degree lenses
- Bladder wash for cytology
- Utilization of a continuous-flow resectoscope to maintain bladder at 50% capacity
- Utilization of a video camera
- Complete resection of all lesions with pure cutting current
- Separate biopsy from tumor base
- Random bladder biopsies and prostatic urethral biopsies as indicated for high-grade disease
- Repeat cystoscopic exam following complete resection
- Adjuvant therapy within 6 h, as indicated

Anesthesia during TUR can be either regional or general. We typically prefer spinal anesthesia for all our TUR cases. The advantages of spinal anesthesia are ease in administration and monitoring of the patient intraoperatively, safety, and a decreased risk of an obturator reflex. Furthermore, it facilitates earlier discharge of the patient from the hospital, allowing these procedures to be done as ambulatory day cases.

Prior to resection, a bimanual examination should be performed — ideally under anesthesia with the bladder empty — especially if bladder invasion into deep muscle or fat is suspected. Another bimanual exam should also be performed after TUR. The accuracy of bimanual examination to predict pathological stage is not well established. For superficial bladder tumors, bimanual examination is unlikely to be helpful, but its routine practice should be encouraged. In fact, an additional benefit of bimanual palpation in the male is the opportunity to palpate the prostate. Prostatic abnormalities should be duly noted and investigated further as appropriate.

It is incumbent on the urologist to view the entire bladder mucosa completely. We typically utilize both a 70- and 30-degree lens to evaluate the entire bladder surface fully. We believe that the 70-degree lens provides us with an improved view of the anterior and lateral walls. All abnormal areas should be recorded and then biopsied separately. Furthermore, a complete cystoscopic exam must be repeated at the end of the TUR to ensure that all tumors and abnormal areas have been resected. Following complete cystoscopic examination, a bladder wash for cytology with approximately 100 cc of sterile saline should be performed. Bladder wash has been demonstrated to have a greater sensitivity for high-grade lesions and carcinoma *in situ* (CIS) than voided urine for cytology[10,11]. The diagnosis of CIS requires an experienced eye and, even then, the changes are subtle. Furthermore, it is important to perform the bladder wash only after the entire bladder mucosa has been evaluated to prevent false-positive areas created by the bladder wash irritating the bladder mucosa.

Ideally, TUR should be performed with a continuous-flow resectoscope and a video monitor. One of the most difficult aspects of a TURBT is to judge the proper depth of resection. The surgeon must take into consideration the tumor appearance and the need to obtain muscle, the bladder thickness, any movement of the bladder during resection, and the depth of penetration of the loop into the wall as the current is applied. The surgeon should be cognizant of the bladder volume since muscle is thinnest when distended. We believe that the continuous-flow resectoscope helps to maintain the bladder volume at a stable capacity (approximately 50% full) and at a safe level. Not only does the video monitor magnify the cystoscopic images, but it also is more comfortable for the operating surgeon. Furthermore, the video monitor allows the resecting surgeon the ability to demonstrate abnormal areas and the technique of TURBT to trainees. While cystoscopic simulators have recently been introduced[12,13], we are not aware of any training module that demonstrates the technique of TURBT as well as observing a proficient urologist.

TURBT can be performed either by shaving the lesion from top to bottom or en-bloc[14]. Surgeon preference tends to dictate which method is more often utilized. Since TURBT is both a staging and a therapeutic procedure, it is incumbent for the urologist to obtain muscle in the specimen when performing a TURBT to prevent understaging of the lesion. Multiple studies have demonstrated the risk of understaging bladder cancer to approach 30% even if muscle is present in the original specimen, and even higher if no muscle was present (Table 2). The biological difference between muscle-invasive and nonmuscle-invasive lesions are significant, and the treatment algorithms are thus different. Not completely resecting a tumor or calling a muscle-invasive lesion "superficial" may alter the patient's long-term prognosis. Because of this significant risk of understaging, the Society of International Urology (SIU) Bladder Cancer Consensus Panel recently recommended that a second TURBT be considered for all patients with high-grade Ta or any T1 urothelial carcinoma[5]. To improve the accuracy of our TUR, we always submit a separate biopsy from the tumor base. This allows the pathologist to evaluate directly for the presence or absence of muscle-invasive disease at the base of the tumor. The utility of random bladder biopsies or bladder mapping is frequently debated. Recent SIU guidelines do not recommend random bladder biopsies for patients with known low-grade lesions or with negative cytology[4]. However, for those with high-grade lesions or CIS, random bladder biopsies can often provide useful prognostic information[5]. Random biopsies are specifically indicated for patients with positive cytology and no visible lesions.

**TABLE 2**
**Understaging in Bladder Cancer**

| Series | % Understaged |
|---|---|
| Herr[21] | 49 |
| Dutta et al.[22] | 30 (64)* |
| Brauers et al.[23] | 24 |
| Freeman et al.[24] | 34 |

* When no muscle present in initial TUR specimen.

While TUR is a relatively safe procedure, complications can occur. The two most frequently seen complications are bleeding and bladder perforation[15], both of which are preventable and can be minimized with good surgical technique. Bladder perforation should always be avoided since disseminated urothelial carcinoma can result[16,17,18]. Nieder et al. recently published a prospective study evaluating the perioperative complications secondary to TURBT in a teaching setting[19]. Among 173 consecutive TURBTs performed at an academic medical center, 5.8% of patients experienced a complication related to the TURBT. Bladder perforation and bleeding requiring a transfusion occurred in 3.5 and 2.3% of patients, respectively; rates consistent with other reports. Moreover, we did not find that the most junior residents were disproportionately involved in the complications. In fact, the most senior residents and fellows were more often involved in cases with perforations, likely related to their preferentially operating in the most difficult cases. Hollenbeck et al. recently reviewed the National Surgical Quality Improvement Program (NSQIP) database from 123 Veterans Affairs Hospitals and reported a 90-day mortality rate of 3.3% for those undergoing TUR[3]. It is important to remember that patients undergoing TUR tend to be not only older, but may also have significant comorbidities, especially those related to chronic tobacco use.

## ADJUVANT TREATMENT

Multiple studies and meta-analyses have demonstrated a benefit to adjuvant intravesical chemotherapy following TUR[20]. The recent WHO/SIU Bladder Cancer Consensus Panel gave a Grade A recommendation that a single dose of chemotherapy should be given at the time of TUR (ideally within 6 h, but no more than 24 h) whether or not additional therapy is planned, as long as no bladder perforation has occurred[5]. We typically instill 40 mg, adjuvant Mitomycin C *immediately* after TUR (while the patient is in the recovery room) and allow it to dwell for 1 h. For those patients whose pathology reveals a low-grade lesion, no further treatment is required. For those patients with high-grade lesions or CIS, a course of BCG is usually initiated 4 weeks following TUR.

## CONCLUSIONS

Most bladder cancers are low-grade superficial tumors. However, a significant number of these are high grade and invasive. Accurate staging and grading during initial evaluation and TUR ensures appropriate treatment. TUR should be performed in a timely fashion and under standard conditions. These include use of proper anesthesia with a continuous-flow video resectoscope. The entire bladder must be visualized and all abnormal areas must be resected, with separate biopsies from each tumor’s base, specifically to avoid understaging muscle-invasive disease. Restaging TUR is recommended for all high-grade tumors, particularly if the muscle is not present in the specimen. Immediate instillation of single dose chemotherapy agents such as Mitomycin C following TUR is recommended.
